# Mediation of the Physical Activity and Healthy Nutrition Behaviors of Preschool Children by Maternal Cognition in China

**DOI:** 10.3390/ijerph13090909

**Published:** 2016-09-13

**Authors:** Xianglong Xu, Manoj Sharma, Lingli Liu, Ping Hu, Yong Zhao

**Affiliations:** 1 School of Public Health and Management, Chongqing Medical University, Chongqing 400016, China; xianglong1989@126.com (X.X.); 13110265315@163.com (L.L.); m18323165365@163.com (P.H.); 2 Research Center for Medicine and Social Development, Chongqing Medical University, Chongqing 400016, China; 3 Collaborative Innovation Center of Social Risks Governance in Health, Chongqing Medical University, Chongqing 400016, China; 4 Behavioral & Environmental Health, Jackson State University, Jackson, MS 39213, USA; manoj.sharma@jsums.edu

**Keywords:** maternal, preschool children, cognition, health behavior, physical activity, screen time

## Abstract

(1) Objective: We aimed to explore the role of social cognitive theory (SCT) of mothers in the physical activity and healthy nutrition behaviors of preschool children; (2) Methods: We used a self-administered five-point Likert common physical activity and nutrition behaviors scale in Chinese based on a social cognitive theory scale in English with established validity and reliability in the USA. The current study adopted the proportional sampling method to survey mothers of preschool children in four areas—namely, Chongqing, Chengdu, Taiyuan, and Shijiazhuang—of China; (3) Results: We included 1208 mothers (80.0% mothers of normal weight children, age 31.87 ± 4.19 years). Positive correlations were found between maternal social cognition and preschool children’s physical activity (PA) behavior (*p* < 0.0001). However, an insignificant correlation is observed between preschool children’s fruits and vegetables (FV) behavior, screen time (ST) behavior, and maternal social cognition; (4) Conclusions: This study provides some implications for increasing fruit and vegetable consumption, increasing physical activity time, and reducing screen time in preschool children using SCT in China. Maternal social cognition is associated with preschool children’s PA behavior, and the results suggest that maternal social cognition may not affect children FV and ST behaviors. Further research is necessary to test the mediation of maternal social cognition on preschool children’s ST behavior and the correlations between maternal social cognition and children’s ST behavior.

## 1. Introduction

Childhood obesity is currently among the most serious public health challenges worldwide. In 2011, more than 40 million children under the age of five were overweight [1]. In China, the overall prevalence of overweight and obesity in children aged two to six years old has been 10.7% and 4.2%, respectively, in the first few years of the 21st century [2]. The prevalence of overweight or obese children has increased at an alarming rate in both developing and developed countries; the age of the onset of childhood obesity has also been gradually decreasing [3]. Given the high risk of obesity and chronic non-communicable diseases later in life, obese children experience adverse outcomes such as breathing difficulties, increased risk of fractures, hypertension, early symptoms of cardiovascular disease, insulin resistance, and psychological effects [4,5,6]. With the development of the Chinese national economy and the improvement of living standards, preschool childhood obesity has become an increasingly serious public health problem in China [7].

The preschool period is an important stage in a child’s development, specifically concerning fat accumulation and obesity. However, preschool obesity is not only related to external factors but also to lifestyle, living environment, and chronic disease [8]. The preschool stage is also the most important period for cognitive and behavioral habit formation and is the best stage for the cultivation and establishment of proper nutrition consciousness, good eating behavior, and a healthy lifestyle. The eating habits and behavior that will be developed at this stage will be carried on until adulthood and will be hard to modify [9]. Research data indicate that obese preschool children are at risk for developing adult obesity and adverse metabolic alterations [10,11]. The factors associated with dietary habits in early childhood may be different from those affecting school-aged children, due to preschoolers’ earlier developmental stage and greater dependence on their family [12].

Most often, mothers are the direct caregivers of children in China. Influenced by traditional ideas, many women in China—particularly housewives—consider domestic service as their primary responsibility; therefore, they have great authority in domestic affairs and focus on the diet and nutrition of their families [13]. Maternal practices such as feeding style (dietary intake), instrumental behavior (e.g., the use of food as a reward), role modeling (particularly weight-related behaviors), and nutritional knowledge have been shown to be associated with a child’s eating and PA habits and weight status [14]. At an early age, children observe their parents’ eating behaviors and typically eat what their parents eat [15]. Numerous studies have shown that mothers play a key role on children’s dietary and exercise habits, particularly in the childhood orientation of dietary and exercise habits [16,17].

Studies have suggested that social cognitive theory offers a practically useful framework for designing primary prevention interventions that will reduce childhood obesity [18]. Social cognitive theory is an effective model for exploring the influential constructs of health behavior [19]. A previous study shows that fruits and vegetables (FV), physical activity (PA), and screen time (ST) (television/video games) are the modifiable lifestyle risk factors for childhood obesity [20]. FV are good for children to control weight, previous studies showed that low-energy dense foods, FV, and a healthy breakfast are negatively associated with overweight and obesity [21,22]. The mothers’ lack of knowledge on the field is also the major cause of children’s bad habits [23]. However, little reliable evidence on physical activity and healthy nutrition behaviors were from China. Little is known about the role of social cognitive theory of mothers on physical activity and healthy nutrition behaviors of preschool children among Chinese population. Studies on the association between maternal cognition and preschool children physical activity and healthy nutrition behaviors are lacking in China. Culture and environment can exert an important influence on social cognitive theory. Improved understanding of the social influences that impact physical activity and healthy nutrition behaviors may lead to the development of educational and environmental strategies that could impact risky preschool children’s behaviors. On the basis of this study, we plan to obtain evidence on the role of social cognitive theory of mothers on the physical activity and healthy nutrition behaviors of preschool children and provide a scientific basis for childhood obesity prevention and control.

## 2. Methods 

### 2.1. Study Population and Design

Study participants were mothers who have three-year-old to seven-year-old children. The current study adopted the proportional sampling method to survey mothers with preschool children in four areas, namely, Chongqing, Chengdu, Taiyuan, and Shijiazhuang in July 2013. The mother’s inclusion criteria include the following: (1) mothers with children aged three-years-old to seven-years-old; (2) children have no health problems that will prevent them from walking, running, or jumping; (3) children are currently not taking any prescribed medication(s) on a long-term basis; (4) children are not in any type of health program designed to help them lose or maintain their current weight; (5) physicians have not prescribed a special diet or additional calories for the child. In each study site, a convenience sampling of mothers with preschool children was included. The sampling sites only included the kindergarten and children’s playgrounds in each city.

According to [24], the rate of maternal cognition on preschool children’s FV eating behavior is about 34.1%. We set P=0.34; Q=1−P=1−0.34=0.66; margin of error $d=0.10P=0.10\times 0.34=0.034,Z_{\alpha }=1.96$. The sampling size is $N=\frac{Z_{\alpha }^{2}\times pq}{d^{2}}=\frac{1.96^{2}\times 0.34\times 0.66}{0.034^{2}}=746$. The total sample size was 1254 in the survey.

### 2.2. Ethical Approval 

This study was approved by the Ethics Committee of Chongqing Medical University (2016001). A written informed consent was obtained from all participants.

### 2.3. Measures

We used a self-administered five-point Likert questionnaire, which was based on social cognitive theory and was specifically designed for mothers with preschool children. A questionnaire was completed after repeated discussions with experts and after the completion of the pilot investigation. The questionnaire included the following sections: mother’s inclusion criteria, child’s behavior status (including PA, FV, and ST) in the past 24 h; mothers’ basic information (age (years), height, weight, marital status (unmarried/married/living with a partner/separated), employment status (employed part-time/employed full-time/unemployed), working hours per week (hours)). BMI is a widely accepted measure for identifying children and adolescents with excess weight [25]. Children whose BMI were at the 10th percentile or lower were defined as underweight, those with BMI greater than the 10th percentile but less than the 90th percentile were assigned to normal weight, and those with BMI equal to or greater than the 90th percentile were defined as overweight [23]. Constructs of maternal cognition questions on the physical activity and healthy nutrition behaviors include the following: (1) maternal cognition on child’s PA behaviors; (2) maternal on child’s FV eating habits behaviors; (3) maternal cognition on child’s ST behaviors.

### 2.4. Survey Implementation 

We first identified the four cities to be used in this study (i.e., Chongqing, Chengdu, Taiyuan, and Shijiazhuang). The kindergarten and children’s playgrounds were selected randomly in each city. At each survey location, participants were selected randomly and asked politely if they would be willing to participate in the campaign. Those who agreed were interviewed face to face by investigators to answer every designed question. The interview time lasted approximately 20 min for each participant. Finally, we collected 1254 participants that conformed to the requirements of the study in July 2013. Among 1254 mothers, 1208 (96.3%) persons answered all questions, for a total of 1208 in the analysis.

### 2.5. Statistical Analyses

Descriptive statistics were used to analyze the data. Scores for each question range from one to five, and a higher score indicates a higher level. ANOVA testing was used to compare differences in continuous variables in three weight status groups. A series of simple regression models was used to investigate the association between maternal social cognition and preschool children health behavior. All statistics were performed using 2-sided tests, and statistical significance was considered at *p* < 0.05. All data analyses were performed using statistical software (SAS version 9.1; SAS Institute, Cary, NC, USA).

## 3. Results

### 3.1. Characteristics of the Participants

The survey included items that would assess the socio-demographic characteristics of mothers and children. The application of the exclusion criteria reduced the target sample size to 1208, which represents 96.3% of the target 1254 interviewees. The mothers’ age of overweight and obese children, normal weight children, and underweight children were 30.79 ± 3.66, 31.87 ± 4.19, and 31.94 ± 3.45, respectively. Significant differences with respect to mothers’ age (*p* = 0.0208), marital status (*p* = 0.0011), employment status (*p* < 0.0001), and working hours per week (*p* = 0.0162) were observed. Furthermore, no statistically significant difference was seen regarding their children’s age and mode of delivery (Table 1).

### 3.2. Preschool Children’s Physical Activity and Nutrition Behaviors Status 

Table 2 shows the differences in preschool children health behavior. The mean servings of fruit that children ate among the overweight and obese groups were significantly more than the underweight group (*p* = 0.0214). The time of child’s PA throughout the day among the overweight and obese groups were significantly longer than that of the normal weight group and underweight group (*p* = 0.0292). The time of child’s PA from structured activities among the overweight and obese groups was significantly shorter than that of the normal weight group and that of the underweight group (*p* = 0.0091). The time of child’s PA from unstructured activities among the overweight and obese groups was significantly longer than that of the normal weight group and the underweight group (*p* < 0.0001). The amount of time spent on a computer by the children in the overweight and obese groups was significantly shorter than that of the normal weight group and the underweight group (*p* = 0.0014). The time children spent playing video games among overweight and obese groups was significantly shorter than that of the normal weight group and the underweight group (*p* = 0.0051) (Table 2).

### 3.3. Maternal Cognition on Preschool Children’s PA Behavior

Table 3 shows the differences on preschool children’s PA behavior. Concerning the score of “how often do you participate in PA with your child at home,” the underweight group was significantly higher than normal weight group (*p* = 0.0071). The score of “encourage your child to be physically active in your home, yard, or apartment complex” among underweight group was significantly higher than normal weight group and overweight and obese groups (*p* < 0.0001). The score of “find fun ways for your child to be physically active in your home, yard, or apartment complex” among overweight and obese groups was significantly less than the normal weight group and underweight group (*p* < 0.0001). The score of “participate in PA with your child in your home, yard, or apartment complex” in overweight and obese groups was significantly less than among the normal weight group and the underweight group (*p* = 0.0002). Concerning the score of “how sure are you that if your child participates in 60 min of PA every day they will have more fun,” the underweight group was significantly higher than the normal weight group and the overweight and obese groups (*p* = 0.0031). Concerning the score of “if your child participates in 60 min of PA every day they will have a better weight,” overweight and obese groups was significantly less than the normal weight group and underweight group (*p* < 0.0001). The score of “how sure do you think if your child participates in 60 min of PA every day they will have more self-confidence” among underweight group was significantly higher than normal weight group, and overweight and obese groups (*p* = 0.0002).

### 3.4. Maternal Cognition on Preschool Children’s FV Eating Behavior

Table 4 summarizes the comparison of preschool children’s FV behavior. The score of “you could get your child to eat three cups of FV every day” among overweight and obese groups was significantly less than that among the normal weight group and underweight group (*p* = 0.0264). However, no significant differences were observed in many aspects on maternal cognition on preschool children’s FV eating behavior among three children’ weight status groups.

### 3.5. Maternal Cognition on Preschool Children’s ST Behaviors

The score of “participate in ST at home with your child” among overweight and obese groups was significantly less than that of the normal weight group and underweight group (*p* = 0.0029). The score of “encourage your children to reduce ST at home” was significantly different among overweight and obese groups and normal weight group (*p* = 0.0095). The score of “find alternative activities to ST” among overweight and obese groups was significantly less than that among the normal weight group and that of the underweight group (*p* = 0.0108). The score of “manage any negative emotions while reducing ST” in overweight and obese groups was significantly less than that of the normal weight group and underweight group (*p* = 0.0153) (Table 5).

### 3.6. Linear Regression for Maternal Cognition and Preschool Children’s Health Behavior

Spearman’s correlation coefficient between maternal cognition and preschool children’s PA behavior in a sample of preschool children was 0.19287, *p* < 0.0001. Spearman’s correlation coefficient between maternal cognition and preschool children’s FV behavior in a sample of preschool children was 0.03874, *p* = 0.1786. Spearman’s correlation coefficient between maternal cognition and preschool children’s ST behavior in a sample of preschool children was −0.02082, *p* = 0.4699. The obese and overweight groups (R^2^ = 0.0339) and underweight group (R^2^ = 0.0342) could be influenced by the mother’s cognition of eating FV compared with the normal weight group (R^2^ = 0.0009). Furthermore, the difference was statistically significant with the obese and overweight groups (*p* = 0.0441) and the underweight group (*p* = 0.0414). However, the normal weight group has a negative result (*p* = 0.3455). The obese and overweight groups (R^2^ = 0.0319) had a small difference between the normal weight group (R^2^ = 0.0385) on explaining the cognitive status. The obese and overweight groups (*p* = 0.0509) had no statistical significance on the regression coefficient, whereas the normal weight group (*p* < 0.0001) and underweight group (*p* = 0.0011) had a statistical significance on the regression coefficient. The mediation of maternal cognition on preschool children’s ST behavior in different weight levels was statistically the same. The R^2^ value of the three groups were low, and no statistical significant meaning explained the linear relationship of the regression equation among the obese and overweight groups (*p* = 0.4581), normal weight group (*p* = 0.3180), and underweight group (*p* = 0.4700).

## 4. Discussion

Furthermore, we found that maternal employment status (full-time, part-time, or unemployed) and work hours affect children’s healthy lifestyle and the family food environment in varying degrees [26,27]. This study shows that unemployed and employed part-time mothers presented the low proportion of overweight and obese infants. In China, unemployed mothers are engaged as full-time housewives and they have enough time to take care of children being full time compared to the busy working mothers, so that could be the reason unemployed mothers presented the lowest proportion. China is a developing country; especially the unemployed groups in central or western (underdeveloped region of China) have limited social security systems, the food supply of those was easily affected, maybe cannot easily buy high energy food, and are less likely to eat excess nutrients. In contrast, European countries are developed countries, the food supply of those were unemployed is not affected by and large, it is even easier to buy high-energy food and more easily have excess nutrients.

This study shows that overweight and obese children eat more fruits than underweight children. However, no significant differences were observed in many aspects on maternal cognition on preschool children’s FV eating behavior among three children’ weight status groups. Studies have shown that mothers become less active in encouraging children to eat FV when their child’s body weight increases [23]. In our survey, compared with the mothers of normal and underweight weight children, the mothers of the overweight and obese children have less confidence in ensuring their children to eat FV every day. A possible reason is that this maternal cognition does not necessarily translate into health behavioral outcomes on their preschool children’s FV eating behavior. Further research will be needed to confirm this. Previous study showed that accessibility and availability of foods at home have an influence on the types of foods children consume. FV take the place of the unhealthy foods at home and can be a better way to force children to consume FV [28].

Our findings found that, compared with children of normal or low weight, overweight and obese children prefer long exercises. However, the time cost of overweight and obese children in participating in structured activities is short. Our study is not similar with other studies that suggest that children who have higher BMIs need longer exercise times [29]. The reasons for these differences may be that children eat too much high energy food and are conscious of being overweight; thus, they will do physical activities. Our research shows that the time spent together by the underweight preschool children and their mothers in physical exercise is longer than that of normal weight children, encouraging children to participate in physical exercise as well. Prior studies have shown that mothers who encourage children to exercise can affect children’s behavior [30]. Some mothers would encourage their children to do physical activities or do physical activities with their children personally when they have time [30]. We consider that mothers engaging their children in sports can increase the preschoolers’ movement time and opportunity, and has a certain effect on children’s weight control. Allowing children with more time to play or do outdoor activities will help children increase PA levels [20]. Considering mothers’ cognition on children’s PA, the mothers of obese and overweight children are less likely to have good cognitive results than the mothers of normal weight children. Previous study showed that the intervention attitude and intensity of mothers’ awareness to campaign has an impact on children’s weight [28]. We can use community, schools, and institutions to conduct health education for mothers; which will increase their awareness and will improve the strength of their children. Further research can be studied indoor or outdoor sports willingness among preschool children, and enhance the enthusiasm and initiative of children’s sports.

Our findings found that the time that overweight and obese children spend on computer (including playing computer games) is shorter than normal and underweight children. If ST is too long, the children will spend more time on screen and will be less likely to participate in PAs. Previous research suggests that mothers’ cognitions are important determinants of children’s ST use [31]. Our study shows that the time spent on the Internet among underweight children is longer than normal weight children. Therefore, we infer that weight is associated with the time spent on surfing the Internet. Furthermore, insignificant differences are observed among preschoolers on ST behavior. Educational value is the most important reason for television and computer use, the children enjoy the opportunity to get things done with their parents [17], but these reasons should not be greater than ST contact-related health effects [32]. Mothers of the overweight and obese children group should be careful about the ST of children [11]. Moreover, the longer children sit in front of the screen, the more possible for a child to eats snacks, thus making children gain more body weight [33]. Therefore, there were diverse ways that affect mother’s regulations of overweight and obese children’s ST [34]. We propose increasing mothers’ perception, such as providing mothers with suggestions about controlling children’s weight and guiding mothers to improve the status of children’s ST. We further recommend that mothers should have strict supervision to reduce the children’s time in facing the screen, while controlling the children to reduce ST. In addition, mothers should spend more time with children playing outdoor sports.

## 5. Strength and Limitations

The present study has several strengths. First, this study further confirms or expands that maternal cognition was associated with preschool children’s PA behavior. Second, this research shows that an insignificant correlation is found between preschool children’s physical activity and healthy nutrition behaviors and maternal social cognition in China. Third, this research also shows an insignificant correlation is also observed between preschool children’s ST behavior and maternal cognition. Forth, the results of this study can aid other countries or regions in implementing health education, as well as provide a reference for further investigations on health education. What’s more, this study included a large number of mothers sample.

This study also has certain limitations. The primary limitation being the lack of objective measurement of the behaviors studied. Second, the cross-sectional survey data reduces the researcher’s ability to make direct causal inferences, to explore whether unmeasured factors may better explain the observed relationships, and to determine the direction of causality. Precise causal inferences require census and cohort studies. Third, our sample was not nationally representative, in terms of potential biases, and the sample consisted of mothers with preschool children in four regions, namely Chongqing and Chengdu in South China, and Taiyuan and Shijiazhuang in North China. Since the sampling sites only included the kindergarten and children’s playgrounds in each city, it lacked random selection of participants which introduced sampling bias. Ideally, a random selection of participants would have added confidence to the study results. Fourth, this result may be the cause of China’s social particularity, and further research will be needed in other areas, particularly in the social attributes of different countries. Fifth, this study has no data regarding mothers’ BMI. Previous study showed that overweight and obese parental weight statuses were both associated with a significantly higher prevalence of child and adolescent obesity [35]. Overweight and obese mothers with accurate perceptions of their own weight status, however, were more likely to have accurate perceptions of their overweight or obese children. If a mother does not identify her child as overweight or obese, she is less likely to support measures to modify lifestyle behaviors to prevent further weight gain [36]. Further research is necessary to consider this. Furthermore, this study did not include gender; and this study aimed to explore the role of social cognitive theory of mothers on the physical activity and healthy nutrition behaviors of preschool children. Therefore, this study cannot get the prevalence of obesity of preschool children.

## 6. Conclusions

It is evident from this study that maternal social cognition plays a role in physical activity and healthy nutrition behaviors of preschool children in China. Health education programs will be needed to improve maternal social cognition on physical activity and healthy nutrition behaviors. This study provides some implications for increasing fruit and vegetable consumption, increasing physical activity time, and reducing screen time in preschool children using SCT of in China. Maternal social cognition is associated with preschool children’s PA behavior, and the results suggest that maternal social cognition may not affect children’s FV and ST behaviors. Further research is necessary to test the mediation of maternal social cognition on preschool children’s ST behavior and the correlations between maternal social cognition and children’s ST behavior.

## Figures and Tables

**Table 1:** Descriptive statistics for Participant by children’s weight status (Mean ± SD/%).

Characteristics	Overweight and Obese (*n* = 120)	Normal Weight (*n* = 966)	Underweight (*n* = 122)	*p*-Value
Maternal characteristics				
Age (years)	30.79 ± 3.66	31.87 ± 4.19	31.94 ± 3.45	0.0208 *
Marital status				
*Unmarried*	0.00%	55.00%	45.00%	0.0011 **
*Married*	10.09%	80.27%	9.65%	
*Living with a partner*	13.04%	86.96%	0.00%	
*Separated*	8.57%	80.00%	11.43%	
Employment status				<0.0001 **
*Employed part-time*	5.53%	88.94%	5.53%	
*Employed full-time*	13.25%	76.56%	10.19%	
*Unemployed*	5.59%	81.25%	13.16%	
Working hours per week (hours)	28.29 ± 15.84	25.63 ± 18.91	21.48 ± 20.31	0.0162 *
Child’s age (years)	5.18 ± 0.84	5.04 ± 0.87	5.13 ± 0.80	0.1724
Mode of delivery				0.5327
*Eutocia*	9.79%	80.67%	9.55%	
*Caesarean Section*	10.00%	78.61%	11.39%	
*Dystocia*	20.00%	70.00%	10.00%	

Note: Significance levels shown are * *p* < 0.05, ** *p* < 0.01.

**Table 2:** Physical activity and nutrition behaviors of preschool Children by weight status (Mean ± SD).

Physical Activity and Nutrition Behaviors	Overweight and Obese (*n* = 120)	Normal Weight (*n* = 966)	Underweight (*n* = 122)	*p*-Value
**Yesterday, how many did your child**				
**FV intake behaviors**				
Servings of eat fruits	2.21 ± 1.04	1.99 ± 1.22	1.79 ± 1.08	0.0214 *
Servings of eat vegetables	1.89 ± 1.12	1.92 ± 1.09	2.07 ± 1.09	0.3640
**PA behaviors**				
Minutes spend on PA throughout the day	113.27 ± 72.91	101.25 ± 53.25	95.04 ± 50.70	0.0292 *
Minutes spend on PA came from structured activities	31.23 ± 42.28	41.05 ± 34.74	43.69 ± 33.31	0.0091 **
Minutes spend on PA came from unstructured activities	82.04 ± 66.75	59.55 ± 45.29	52.79 ± 34.47	<0.0001 **
**ST behaviors**				
Minutes spend on watch TV, DVDs, or movies	75.75 ± 59.22	88.26 ± 60.41	82.91 ± 56.07	0.0768
Minutes spend on a computer	22.51 ± 32.12	34.10 ± 33.02	33.28 ± 33.05	0.0014 **
Minutes spend on playing video games	13.58 ± 30.12	22.57 ± 28.61	21.84 ± 26.22	0.0051 **

Note: (1) a serving: a small apple, a banana, 16 grapes, and a pear. The following said half the serving: half a medium melons, a big plum, a medium-sized watermelon disc; (2) Beverage and water: Each 200 mL is a serving; (3) Significance levels shown are * *p* < 0.05, ** *p* < 0.01.

**Table 3:** Maternal Social Cognitive on Preschool Children’s PA Behaviors by children’ weight status (Mean ± SD).

Scale: Grade 1–5	Overweight and Obese (*n* = 120)	Normal Weight (*n* = 966)	Underweight (*n* = 122)	*p*-Value
**How often do you**				
Find fun ways for your child to be physically active in your home, yard, or apartment complex	3.80 ± 1.27	3.59 ± 1.05	3.66 ± 0.95	0.1008
Participate in PA with your child in your home, yard, or apartment complex	3.74 ± 1.15	3.58 ± 0.99	3.85 ± 0.81	0.0071 **
Encourage your child to be physically active in your home, yard, or apartment complex	3.24 ± 1.23	3.48 ± 1.03	3.82 ± 0.93	<0.0001 **
**How sure are you**				
Find fun ways for your child to be physically active in your home, yard, or apartment complex	3.01 ± 1.35	3.44 ± 1.10	3.70 ± 0.96	<0.0001 **
Participate in PA with your child in your home, yard, or apartment complex	2.99 ± 1.45	3.43 ± 1.05	3.42 ± 0.95	0.0002 **
Encourage your child to be physically active in your home, yard, or apartment complex	3.47 ± 1.23	3.51 ± 0.98	3.57 ± 0.94	0.6998
**How confident are you**				
Physically active for 60 min every day	3.48 ± 1.29	3.43 ± 0.99	3.48 ± 0.97	0.8030
Physically active for 60 min every day, even if he or she is tired	3.48 ± 1.24	3.33 ± 1.08	3.47 ± 1.01	0.2187
Physically active for 60 min every day, even if you are tired	3.04 ± 1.27	3.31 ± 1.12	3.25 ± 0.98	0.0450 *
**How sure are you**				
If your child participates in 60 min of PA every day they will				
Get sick less often	3.86 ± 1.12	3.80 ± 1.05	4.02 ± 0.96	0.0852
Have more fun	3.59 ± 1.34	3.76 ± 1.06	4.05 ± 0.82	0.0031 **
Have a better weight	3.34 ± 1.46	3.79 ± 1.11	3.94 ± 0.95	<0.0001 **
Have more self-confidence	3.44 ± 1.42	3.75 ± 1.10	4.03 ± 0.91	0.0002 **

Note: (1) Significance levels shown are * *p* < 0.05, ** *p* < 0.01; (2) How often do you: Never-Always: scores range from 1 to 5 and a higher score indicates the frequency is higher; (3) How sure are you: Not At All Sure-Completely Sure 1–5: scores range from 1 to 5 and a higher score indicates the mothers are surer of their behavior; (4) How confident are you: Not At All Confident-Completely Confident 1–5: scores range from 1 to 5 and a higher score indicates the mothers are more confident of their behavior.

**Table 4:** Maternal Social Cognitive on Preschool Children’s FV Behaviors by children’ weight status.

Scale: Grade 1–5	Overweight and Obese (*n* = 120)	Normal Weight (*n* = 966)	Underweight (*n* = 122)	*p*-Value
**How often do you**				
Have enough FV at home for your child to be able to eat five cups every day	3.53 ± 1.28	3.50 ± 1.04	3.63 ± 1.00	0.4522
Eat at least five cups of FV with your child at home every day	3.44 ± 1.25	3.36 ± 1.12	3.42 ± 1.03	0.6668
Encourage your child to eat five cups of FV at home every day	3.23 ± 1.36	3.43 ± 1.09	3.39 ± 0.97	0.1617
**How sure are you**				
Have enough FV at home for your child to be able to eat five cups every day	3.53 ± 1.28	3.50 ± 1.04	3.63 ± 1.00	0.4522
Eat at least five cups of FV with your child at home every day	3.44 ± 1.25	3.36 ± 1.12	3.42 ± 1.03	0.6668
Encourage your child to eat five cups of FV at home every day	3.23 ± 1.36	3.43 ± 1.09	3.39 ± 0.97	0.1617
**How confident are you**				
Eat three cups of FV every day	3.21 ± 1.31	3.44 ± 1.02	3.56 ± 0.97	0.0264 *
Eat three cups of FV every day, even if they do not like them	3.35 ± 1.33	3.35 ± 1.04	3.44 ± 1.02	0.6838
Eat three cups of FV every day, even if others in the family do not like them	3.31 ± 1.27	3.32 ± 1.11	3.37 ± 1.07	0.8910

Note: (1) Significance levels shown are * *p* < 0.05; (2) How often do you: Never-Always: scores range from 1 to 5 and a higher score indicates the frequency is higher; (3) How sure are you: Not At All Sure-Completely Sure 1–5: scores range from 1 to 5 and a higher score indicates the mothers are surer of their behavior; (4) How confident are you: Not At All Confident-Completely Confident 1–5: scores range from 1 to 5 and a higher score indicates the mothers are more confident of their behavior.

**Table 5:** Maternal Social Cognitive on Preschool Children’s ST behaviors by children’ weight status.

Scale: Grade 1–5	Overweight and Obese (*n* = 120)	Normal Weight (*n* = 966)		Underweight (*n* = 122)	*p*-Value
**How often do you**					
Participate in ST at home with your child	3.18 ± 1.24		3.46 ± 1.01	3.62 ± 0.96	0.0029 **
Encourage your child to reduce ST at home	3.22 ± 1.32		3.50 ± 1.03	3.34 ± 0.90	0.0095 **
Find alternatives to ST at home for your child	3.28 ± 1.36		3.47 ± 1.05	3.50 ± 1.05	0.1570
**How sure are you**					
Adjust to reducing ST to less than 2 h per day	3.18 ± 1.24		3.46 ± 1.01	3.62 ± 0.96	0.2879 *
Find alternative activities to ST	3.22 ± 1.32		3.50 ± 1.03	3.34 ± 0.90	0.0108 *
Manage any negative emotions while reducing screen time	3.28 ± 1.36		3.47 ± 1.05	3.50 ± 1.05	0.0153 *
**How confident are you**					
Spend no more than 2 h per day with ST	3.19 ± 1.26		3.39 ± 1.01	3.46 ± 0.97	0.0907
Reduce ST even if their favorite show is coming on	3.13 ± 1.26		3.27 ± 1.10	3.10 ± 1.01	0.1546
Reduce ST even if everyone else in the family is watching	3.29 ± 1.21		3.21 ± 1.15	3.27 ± 1.21	0.6716

Note: (1) Significance levels shown are * *p* < 0.05, ** *p* < 0.01; (2) How often do you: Never-Always: scores range from 1 to 5 and a higher score indicates the frequency is higher; (3) How sure are you: Not At All Sure-Completely Sure 1–5: scores range from 1 to 5 and a higher score indicates the mothers are surer of their behavior; (4) How confident are you: Not At All Confident-Completely Confident 1–5: scores range from 1 to 5 and a higher score indicates the mothers are more confident of their behavior.

## References

[1] WHO (2013). WHO Media Centre: Obesity and Overweight. http://www.who.int/mediacentre/factsheets/fs311/en/.

[2] Jiang J., Rosenqvist U., Wang H., Greiner T., Ma Y., Toschke A.M. (2006). Risk factors for overweight in 2 to 6-year-old children in Beijing, China. Int. J. Pediatr. Obes..

[3] Wang Y., Lobstein T. (2006). Worldwide trends in childhood overweight and obesity. Int. J. Pediatr. Obes..

[4] Caprio S., Daniels S.R., Drewnowski A., Kaufman F.R., Palinkas L.A., Rosenbloom A.L., Schwimmer J.B. (2008). Influence of race, ethnicity, and culture on childhood obesity: Implications for prevention and treatment: A consensus statement of Shaping America’s Health and the Obesity Society. Diabetes Care.

[5] Choudhary A.K., Donnelly L.F., Racadio J.M., Strife J.L. (2007). Diseases associated with childhood obesity. Am. J. Roentgenol..

[6] Daniels S.R., Arnett D.K., Eckel R.H., Gidding S.S., Hayman L.L., Kumanyika S., Williams C.L. (2005). Overweight in children and adolescents: Pathophysiology, consequences, prevention, and treatment. Circulation.

[7] Ji C. (2006). Global school-age child and adolescent overweight and obesity epidemic situation and the trend. Chin. J. Sch. Health.

[8] Li H. (2006). Current situation and countermeasures of childhood obesity. Chin. J. Med..

[9] Chen C. (2004). Timely actions on childhood obesity. Chin. J. Epidemiol..

[10] Graversen L., Sorensen T.I., Petersen L., Sovio U., Kaakinen M., Sandbaek A., Laitinen J., Taanila A., Pouta A., Järvelin M.-R. (2014). Preschool weight and body mass index in relation to central obesity and metabolic syndrome in adulthood. PLoS ONE.

[11] Parkes A., Sweeting H., Wight D., Henderson M. (2013). Do television and electronic games predict children’s psychosocial adjustment? Longitudinal research using the UK Millennium Cohort Study. Arch. Dis. Child.

[12] Pearson N., Biddle S.J., Gorely T. (2009). Family correlates of fruit and vegetable consumption in children and adolescents: A systematic review. Public Health Nutr..

[13] Saaka M. (2014). Relationship between mothers’ nutritional knowledge in childcare practices and the growth of children living in impoverished rural communities. J. Health Popul. Nutr..

[14] Skouteris H., McCabe M., Swinburn B., Newgreen V., Sacher P., Chadwick P. (2011). Parental influence and obesity prevention in pre-schoolers: A systematic review of interventions. Obes. Rev..

[15] Oliveria S.A., Ellison R.C., Moore L.L., Gillman M.W., Garrahie E.J., Singer M.R. (1992). Parent-child relationships in nutrient intake: The Framingham Children’s Study. Am. J. Clin. Nutr..

[16] Dabelea D., Crume T. (2011). Maternal environment and the transgenerational cycle of obesity and diabetes. Diabetes.

[17] Dowda M., Pfeiffer K.A., Brown W.H., Mitchell J.A., Byun W., Pate R.R. (2011). Parental and environmental correlates of physical activity of children attending preschool. Arch. Pediatr. Adolesc. Med..

[18] Sharma M., Wagner D.I., Wilkerson J. (2005). Predicting childhood obesity prevention behaviors using social cognitive theory. Int. Q. Community Health Educ..

[19] Rinderknecht K., Smith C. (2004). Social cognitive theory in an after-school nutrition intervention for urban Native American youth. J. Nutr. Educ. Behav..

[20] Howie E.K., Brown W.H., Dowda M., McIver K.L., Pate R.R. (2013). Physical activity behaviours of highly active preschoolers. Pediatr. Obes..

[21] He K., Hu F.B., Colditz G.A., Manson J.E., Willett W.C., Liu S. (2004). Changes in intake of fruits and vegetables in relation to risk of obesity and weight gain among middle-aged women. Int. J. Obes. Relat. Metab. Disord..

[22] Shah T., Purohit G., Nair S.P., Patel B., Rawal Y., Shah R.M. (2014). Assessment of obesity, overweight and its association with the fast food consumption in medical students. J. Clin. Diagn. Res..

[23] Christian M.S., Evans C.E.L., Conner M., Ransley J.K., Cade J.E. (2012). Study protocol: Can a school gardening intervention improve children’s diets?. BMC Public Health.

[24] Wang W. (2008). Investigation of the nutrition knowledge, attitude and behavior of preschool children’s parents. Modern Prev. Med..

[25] Force U.S. (2010). Preventive Services Task; Barton, M. Screening for obesity in children and adolescents: US Preventive Services Task Force recommendation statement. Pediatrics.

[26] Bauer K.W., Hearst M.O., Escoto K., Berge J.M., Neumark-Sztainer D. (2012). Parental employment and work-family stress: associations with family food environments. Soc. Sci. Med..

[27] Brown J.E., Broom D.H., Nicholson J.M., Bittman M. (2010). Do working mothers raise couch potato kids? Maternal employment and children’s lifestyle behaviours and weight in early childhood. Soc. Sci. Med..

[28] Campbell K.J., Crawford D.A., Salmon J., Carver A., Garnett S.P., Baur L.A. (2007). Associations between the home food environment and obesity-promoting eating behaviors in adolescence. Obesity.

[29] Decelis A., Jago R., Fox K.R. (2014). Physical activity, screen time and obesity status in a nationally representative sample of Maltese youth with international comparisons. BMC Public Health.

[30] O’Dwyer M.V., Fairclough S.J., Knowles Z., Stratton G. (2012). Effect of a family focused active play intervention on sedentary time and physical activity in preschool children. Int. J. Behav. Nutr. Phys. Act..

[31] Smith B.J., Grunseit A., Hardy L.L., King L., Wolfenden L., Milat A. (2010). Parental influences on child physical activity and screen viewing time: A population based study. BMC Public Health.

[32] LeBlanc A.G., Spence J.C., Carson V., Connor Gorber S., Dillman C., Janssen I., Kho M.E., Stearns J.A., Timmons B.W., Tremblay M.S. (2012). Systematic review of sedentary behaviour and health indicators in the early years (aged 0–4 years). Appl. Physiol. Nutr. Metab..

[33] Veldhuis L., Vogel I., Renders C.M., van Rossem L., Oenema A., HiraSing R.A., Raat H. (2012). Behavioral risk factors for overweight in early childhood; the “Be active, eat right” study. Int. J. Behav. Nutr. Phys. Act..

[34] Laurson K.R., Lee J.A., Gentile D.A., Walsh D.A., Eisenmann J.C. (2014). Concurrent associations between physical activity, screen time, and sleep duration with childhood obesity. ISRN Otolaryngol..

[35] Jiang M.H., Yang Y., Guo X.F., Sun Y.X. (2013). Association between child and adolescent obesity and parental weight status: A cross-sectional study from rural North China. J. Int. Med. Res..

[36] Dowd K.P., Kirwan R.P., Hannigan A., Purtill H., O’Gorman C.S. (2016). The association between maternal perceptions of own weight status and weight status of her child: Results from a national cohort study. Arch. Dis. Child..

